# Prevalence of Fracture Risk Factors in Postmenopausal Women Enrolled in the POSSIBLE US Treatment Cohort

**DOI:** 10.1155/2013/715025

**Published:** 2013-03-31

**Authors:** Nicole Yurgin, Sally Wade, Sacha Satram-Hoang, David Macarios, Marc Hochberg

**Affiliations:** ^1^Amgen Inc., One Amgen Center Drive, Thousand Oaks, CA 91320-1799, USA; ^2^Wade Outcomes Research and Consulting, 358 South 700 East, Suite B432, Salt Lake City, UT 84102, USA; ^3^Q.D. Research, Inc., 8789 Auburn Folsom Road, Suite C501, Granite Bay, CA 95746, USA; ^4^Division of Rheumatology and Clinical Immunology, University of Maryland School of Medicine, 10 S. Pine Street, MSTF 8-34, Baltimore, MD 21201, USA

## Abstract

Subject- and physician-reported data from 4,429 postmenopausal women receiving osteoporosis treatment in the Prospective Observational Scientific Study Investigating Bone Loss Experience (POSSIBLE US) were used to assess the prevalence of risk factors (RFs) and on-study fracture. RFs assessed at study entry were age >70 years; fracture since age 50; minimum T-score (hip/spine) ≤−2.5 at diagnosis; body mass index <18.5 kg/m^2^; rheumatoid arthritis; parental history of hip fracture; current smoking; and recent oral glucocorticoid use. Data were collected with semiannual self-administered questionnaires. Results were stratified by physician-reported osteoporosis/osteopenia diagnosis. Low T-score and age >70 years were the most common RFs in the osteoporosis group, and age >70 years and prior fracture were the most common risk factors in the osteopenia group. Multiple RFs were more common than a single RF in osteoporotic women (54.2% versus 34.6%; *P* < 0.0001) but not osteopenic women (13.8% versus 33.6%; *P* < 0.0001). Women with multiple RFs had more on-study osteoporosis-related fractures than women with a single RF (osteoporosis group: 9.9% versus 6.2%; *P* = 0.0092; osteopenia group: 11.2% versus 4.7%; *P* < 0.0001). In postmenopausal women receiving osteoporosis treatment, multiple RFs increased fracture risk. RFs, in addition to bone mineral density, can help identify candidates for osteoporosis treatment.

## 1. Introduction

The WHO fracture risk assessment tool (FRAX) is a computer-based algorithm that assesses fracture probability of men and women [1]. FRAX was developed using clinical risk factor data from population-based cohorts with 250,000 person-years of followup from Europe, North America, and Japan [2]. This approach uses easily obtained information on clinical risk factors to estimate the 10-year risk of hip fracture and major osteoporotic fracture (spine, forearm, hip, or shoulder) and was incorporated into the United States (U.S.) Preventive Services Task Force osteoporosis screening recommendations in 2011 [3]. The National Osteoporosis Foundation (NOF) also recommends that physicians use FRAX when possible, along with a detailed medical history, physical examination, and bone mineral density (BMD) assessment to diagnose osteoporosis and guide treatment decisions [4]. However, little is known about the prevalence of specific fracture risk factors in postmenopausal women who are currently being treated for osteoporosis.

In the current study, the prevalence of risk factors for fracture in a treated population was assessed using data from the Prospective Observational Scientific Study Investigating Bone Loss Experience in the US (POSSIBLE US), which was a large, longitudinal cohort study of postmenopausal women who were prescribed osteoporosis therapy in a primary care setting [5]. We also examined the associations between the number of risk factors per subject at study entry and the incidence of on-study fracture. We computed FRAX scores using the online calculation tool, assessed the distribution of these scores, and examined the on-study fracture experience of women with different risk strata. 

## 2. Methods

### 2.1. Data Source and Study Population

From October 2005 to January 2007, 134 primary care physicians in the US enrolled 5,015 postmenopausal women who were receiving treatment for bone loss into the POSSIBLE US treatment cohort. The study design and subject characteristics have been previously described [5]. Briefly, all subjects in this Institutional Review Board-approved study had been identified for osteoporosis therapy by their primary care physician and prescribed ≥1 of the following osteoporosis medications: oral bisphosphonate (i.e., alendronate/alendronate sodium with cholecalciferol, risedronate/risedronate with calcium, and ibandronate); oral or transdermal postmenopausal estrogen; parathyroid hormone; calcitonin; raloxifene; or calcium; and/or vitamin D. Calcium and vitamin D supplements were classified as nonpharmacological therapy. Since this was an observational study of routine care, prescribing decisions were based on the judgment of the enrolling physicians. After providing informed consent at a routine visit with the enrolling primary care physician, each subject completed a self-administered baseline questionnaire to report demographic characteristics and lifestyle behaviors, osteoporosis medication use, satisfaction and side effects related to these osteoporosis therapies, and health-related quality of life. Followup questionnaires (mailed to subjects every 6 months after entry for up to 3 years) also included questions about the occurrence of on-study fracture. For each subject, the enrolling physician provided relevant medical history at both study entry and for routine followup visits.

Women from the POSSIBLE US cohort who had a physician-reported diagnosis of osteoporosis or osteopenia on the study enrollment form (*N* = 4,429) were included in these analyses. Risk factors were identified using physician- and subject-reported data collected at the subject's enrollment into the study and included: age >70 years; history of fracture since age 50; minimum reported hip or spine T-score ≤−2.5 at diagnosis; body mass index <18.5 kg/m^2^; rheumatoid arthritis; parental history of hip fracture; current cigarette smoking; and oral glucocorticoid use in the 6 months prior to study entry. BMD assessments were not required for study enrollment; however, diagnostic T-scores were available for 89% of subjects with osteoporosis and 92% of subjects with osteopenia. 

### 2.2. Statistical Analysis

Descriptive statistics (counts and percentages) quantified the prevalence of risk factors, with results reported separately for subjects diagnosed by their enrolling physician with either osteoporosis (osteoporosis group) or osteopenia (osteopenia group). Statistical differences between groups were assessed using chi-square tests for categorical data. 

Available data were used to calculate the 10-year probability of hip fracture and major osteoporosis-related fracture using the US version of the online FRAX calculation tool [1]. FRAX uses race-specific norms; therefore, FRAX scores were calculated only for POSSIBLE US subjects who reported their race as Caucasian, Hispanic, African American, or Asian. Diagnoses of secondary osteoporosis and relevant alcohol use (≥3 units per day) were defaulted to “no” in the calculation tool for all subjects as these data were not collected in the POSSIBLE US cohort. The distribution of FRAX scores was assessed categorically, and the mean (95% confidence intervals) and median FRAX scores were computed.

Incident fractures reported by subjects throughout follow up were classified as either osteoporosis-related or not osteoporosis-related using a published classification schema [6]. Specifically, fractures occurring at locations listed by Warriner et al. [6] as “more likely to be because of osteoporosis” were considered osteoporosis-related for the purposes of this analysis. The number and percentage of subjects with on-study fracture were reported for all fractures and osteoporosis-related fractures and by fracture location. Fracture incidence was computed separately for subjects with osteoporosis and osteopenia, and results were stratified by the number of risk factors at study entry identified for each subject (0, 1, ≥2). 

Chi-square tests for categorical data were used to compare fracture incidence for women who met different risk thresholds (i.e., ≥3% 10-year predicted risk for hip fracture or ≥20% 10-year predicted risk for major osteoporotic fracture) based on NOF classification of FRAX scores. Statistical significance was assessed using pairwise comparisons of the percentage of subjects with on-study fracture for subjects with scores above and below the risk thresholds. Since FRAX was originally developed and validated in untreated populations, we analyzed data for subjects who were not using pharmacologic therapy on entry into POSSIBLE US (i.e., subjects who reported either not using any osteoporosis agent or taking only calcium/vitamin D at or within 2 months of study entry). An additional analysis was conducted using data from pharmacologically treated subjects (i.e., subjects who reported using pharmacological therapy at or within 2 months of entering the study). Missing data were not imputed, and all statistical analyses were conducted using SAS version 9.1 software (SAS Institute Inc., Cary, NC, USA). 

## 3. Results

Data were analyzed for 1,916 women in the osteoporosis group (age: mean 67.8 years; median 61.0 years) and 2,513 women in the osteopenia group (age: mean 62.2 years; median 61.0 years). The majority of women in each group were Caucasian: 87% in the osteoporosis group and 90% in the osteopenia group. Mean followup was 869 days (median: 959 days) for the osteoporosis group and 873 days (median: 932 days) for the osteopenia group. Among the pharmacologically treated patients in the study population, the probability of persisting with the osteoporosis therapy used at study entry was 66% (95% confidence interval: 64%, 68%) at 12 months after study entry [7].

**Figure 1:**
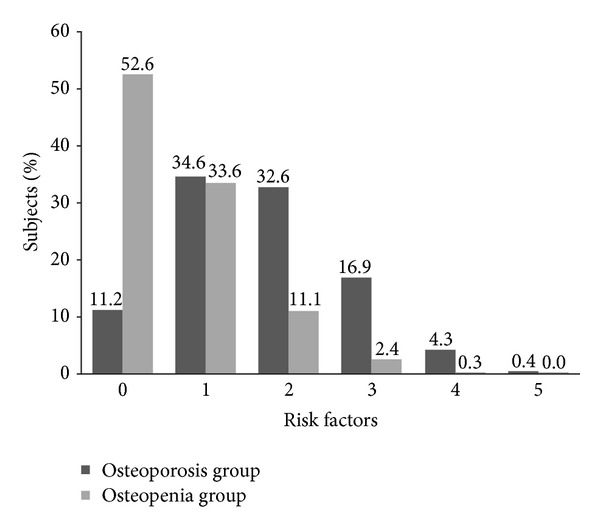
Percentage of subjects with osteoporosis or osteopenia by number of fracture risk factors.

The number of reported risk factors per subject ranged from 0 to 5 (Figure 1). Subjects in the osteopenia group were more likely to have 0 risk factors compared with subjects in the osteoporosis group (52.6% versus 11.2%, *P* < 0.0001). Multiple risk factors were more common than a single risk factor in the osteoporosis group (54.2% versus 34.6%, *P* < 0.0001) but not in the osteopenia group (13.8% versus 33.6%, *P* < 0.0001). 

**Figure 2:**
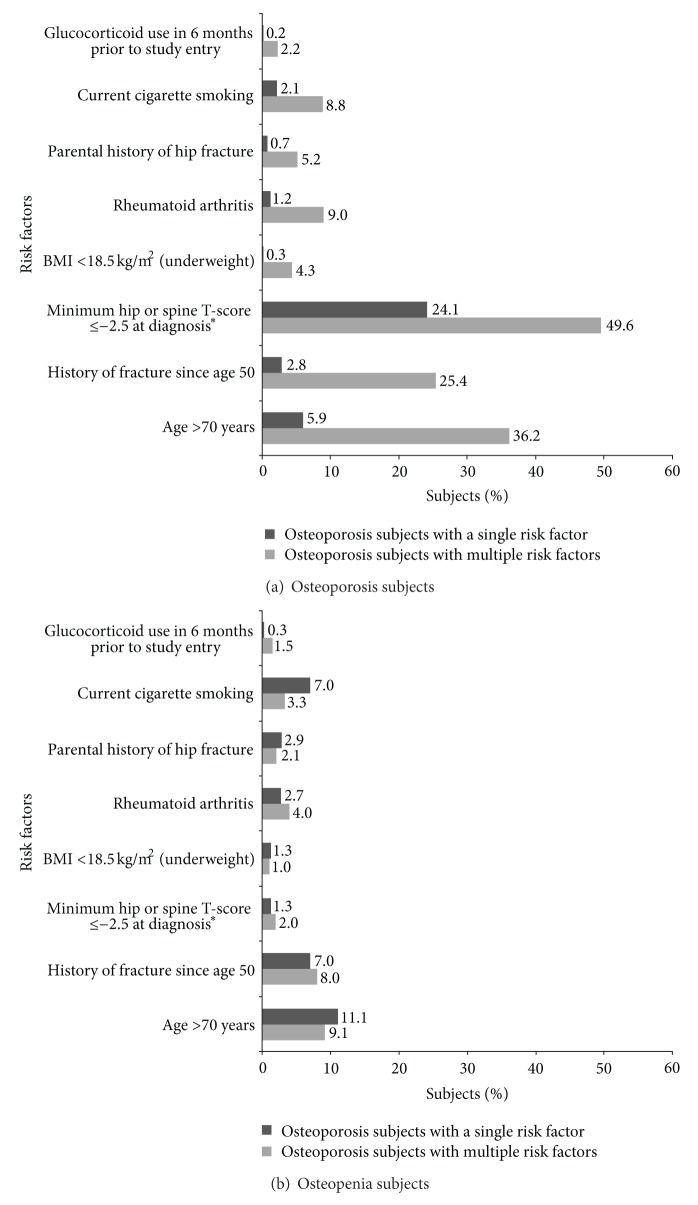
Prevalence of fracture risk factors among subjects with osteoporosis or osteopenia. *Number of subjects with minimum T-scores: 1,703 (88.9%) in osteoporosis subjects; 2,322 (92.4%) in osteopenia subjects. BMI, body mass index.

Among subjects in the osteoporosis group, most common fracture risk factors (singly and in combination) were hip or spine T-score ≤−2.5 at diagnosis, age >70 years, and history of fracture since age 50 (Figure 2(a)). These 3 risk factors were reported in 2.8% to 24.1% of osteoporosis subjects with a single risk factor and in 25.4% to 49.6% of subjects with multiple risk factors.

The most common fracture risk factors among subjects in the osteopenia group who had a single risk factor reported were age >70 years, history of fracture since age 50, and current cigarette smoking, occurring in 11.1%, 7.0%, and 7.0% of subjects, respectively (Figure 2(b)). These 3 risk factors along with rheumatoid arthritis occurred in 3.3% to 9.1% of osteopenia subjects with multiple risk factors.

**Figure 3:**
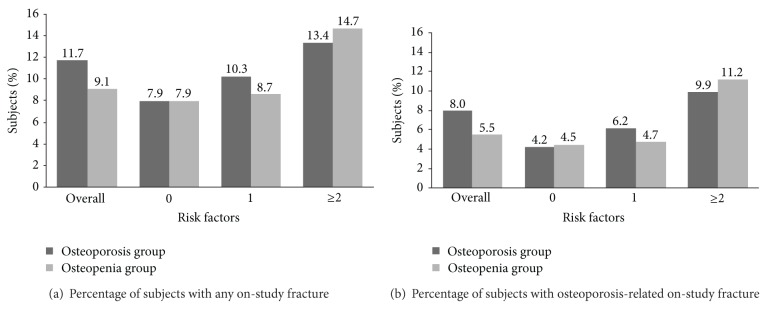
Percentage of subjects reporting on-study fracture stratified by number of fracture risk factors.

Table 1 summarizes subject-reported on-study fracture. At least 1 on-study fracture of any type was reported by 11.7% of women in the osteoporosis group and 9.1% of women in the osteopenia group (*P* = 0.0059). Overall, 8.0% of the osteoporosis group and 5.5% of the osteopenia group (*P* < 0.0001) reported an osteoporosis-related fracture. The highest incidence of on-study fracture and osteoporosis-related on-study fracture occurred in women with multiple risk factors (Figure 3). The percentage of women in the osteoporosis group reporting any on-study fracture ranged from 7.9% for those with 0 risk factors to 13.4% for those with multiple risk factors, and the percentage with osteoporosis-related fractures ranged from 4.2% for those with 0 risk factors to 9.9% for women with multiple risk factors. Corresponding results for the osteopenia group were from 7.9% to 14.7% for any on-study fracture and from 4.5% to 11.2% for osteoporosis-related fracture. On-study osteoporosis-related fractures were more common in women with multiple risk factors compared with women with a single risk factor in both the osteoporosis group (9.9% versus 6.2%, *P* = 0.0092) and the osteopenia group (11.2% versus 4.8%, *P* < 0.0001, Table 2). Osteoporosis-related fractures at locations other than the hip or spine were the most common in both the osteoporosis and osteopenia groups (Table 2). The percentage of subjects with a nonhip, nonspine osteoporosis-related fracture ranged from 3.3% to 6.2% in the osteoporosis group and from 3.6% to 6.9% in the osteopenia group, depending on the number of risk factors. Similarly, the percentage of subjects with multiple osteoporosis-related fractures ranged from 0.5% to 3.4% in the osteoporosis group and from 1.1% to 3.7% in the osteopenia group. 

FRAX scores were computed for 4,295 (97%) subjects in the POSSIBLE US cohort. The mean (95% confidence intervals) 10-year predicted hip fracture risk was 3.8% (3.6%, 4.0%) for subjects pharmacologically treated for bone loss (*n* = 2,996) and 3.3% (3.1%, 3.6%) for nonpharmacologically treated subjects (*n* = 1,299). The median scores for these 2 subject groups were 1.8% and 1.4%, respectively. The mean (95% confidence intervals) 10-year predicted risk for any major osteoporotic fracture was 13.6% (13.3%, 14.0%) for pharmacologically treated subjects and 12.4% (11.9%, 12.9%) for nonpharmacologically treated subjects. 

**Figure 4:**
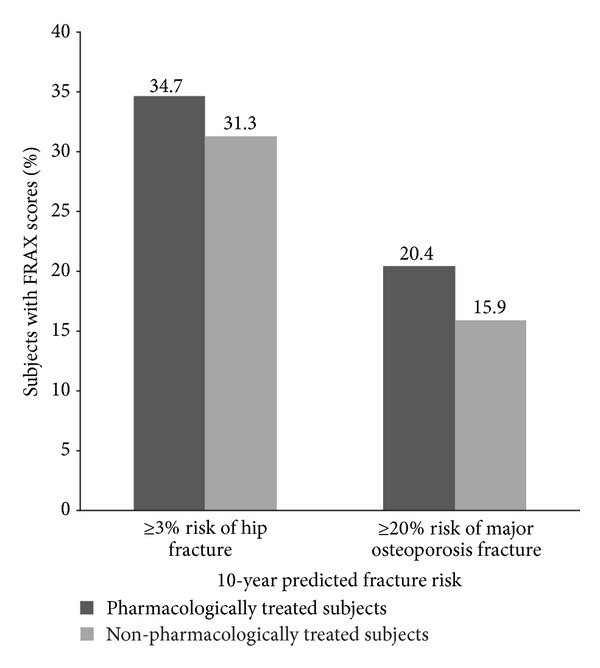
Percentage of subjects in the osteoporosis and osteopenia groups whose FRAX scores met or exceeded the National Osteoporosis Foundation treatment threshold.

A greater percentage of the pharmacologically treated subjects met or exceeded the NOF threshold for major osteoporosis fracture risk compared with nonpharmacologically treated subjects (20.4% versus 15.9%, *P* < 0.001; Figure 4), and this finding also held for the hip fracture risk threshold (34.7% versus 31.3%, *P* = 0.033). On-study fracture incidence was similar for nonpharmacologically treated subjects whose FRAX scores met or exceeded the NOF thresholds for hip fracture or major osteoporotic fracture compared with subjects whose scores were below these thresholds (Table 3). However, similar analyses were conducted for the 2,996 pharmacologically treated subjects with FRAX scores (Table 4), and the incidence of on-study fracture (any and osteoporotic) was significantly higher among subjects whose risk scores met or exceeded the NOF thresholds (*P* < 0.0001). Overall, 5.7% of the 407 subjects who met or exceeded the 3% hip fracture risk threshold experienced an on-study fracture compared with 5.5% of the 892 subjects with scores below the threshold (*P* = 0.95). Of the 206 subjects who met or exceeded the 20% threshold for major osteoporotic fracture risk, 6.8% experienced an on-study fracture compared with 5.3% of the 1,093 whose FRAX scores were below the threshold (*P* = 0.32). There were also no statistically significant differences in the incidence of osteoporosis-related on-study fractures relative to the NOF risk thresholds. 

In the 2,996 pharmacologically treated subjects with FRAX scores (Table 4), the incidence of any on-study fracture was significantly higher among subjects whose risk scores met or exceeded the NOF thresholds (16.7% versus 10.1%, *P* < 0.0001). The same pattern was observed for osteoporosis-related fractures, which occurred in 12.0% of pharmacologically treated subjects who met or exceeded the treatment threshold compared with 5.8% of pharmacologically treated subjects whose FRAX scores were below the NOF threshold (*P* < 0.0001). In combined analysis of pharmacologically treated and nonpharmacologically treated subjects with FRAX scores (*n* = 4,295), 13.6% of the subjects with predicted 10-year hip fracture risk of ≥3% experienced an on-study fracture, and 8.7% of the subjects with predicted hip fracture risk were below this threshold (*P* < 0.0001). Similarly, 16.2% of the subjects with predicted 10-year major osteoporotic fracture risk of ≥20% experienced an on-study fracture. This compares with 5.0% of the subjects who had a predicted risk of major osteoporosis fracture below this threshold (*P* < 0.0001).

## 4. Discussion

The prevalence of key risk factors for fracture has been evaluated in untreated populations in the course of developing and validating FRAX [2], and FRAX was recently validated in a Canadian cohort that included treated individuals [8]; however, this is the first study to our knowledge to examine the prevalence of these risk factors in a cohort of postmenopausal women who have been identified with, and are undergoing treatment for, osteoporosis or low bone mass in the primary care setting in the USA. In this treatment cohort, 1 in 2 osteoporotic women and nearly 1 in 7 osteopenic women had multiple risk factors for fracture. With a median followup period of approximately 2.6 years, 8.0% of women in the osteoporosis group and 5.5% of women in the osteopenia group experienced an osteoporosis-related on-study fracture with the majority (68.0% and 71.7%, resp.,) of these fractures occurring at sites other than the hip or spine in both groups. The on-study fracture incidence was even higher among subjects who met the NOF FRAX treatment thresholds. 

Osteoporosis-related fractures were also significantly more common in women with multiple risk factors compared with women with a single risk factor. Interestingly, women with multiple risk factors had a similar fracture incidence regardless of whether they were in the osteoporosis group or osteopenia group. 

For osteoporosis, effective treatment involves identifying at-risk individuals, determining the likely causes and factors contributing to low bone mass, and tailoring medical treatments and other interventions (e.g., fall prevention) to the individual patient's needs [9]. The results of our study may help inform primary care physicians' approaches to the first component of this treatment paradigm—patient identification. In particular, women of age > 70 years, with history of fracture, and/or current smoking (the most common non-BMD risk factors in this cohort of postmenopausal women) may merit further assessment of their bone health and potential fracture risk. Our results also suggest that women with multiple risk factors may be more likely to experience an osteoporosis-related fracture even after having bone-specific medications prescribed. The greater incidence of fractures among women with multiple risk factors during the relative short observation period (from 2 to 3 years) underscores the importance of determining how many fracture risk factors each patient has and suggests that primary care physicians may want to closely monitor patients with multiple risk factors, even after they initiate therapy.

Published guidelines recommend using risk factor profiles to identify candidates for osteoporosis therapy. As recently as 2003, osteoporosis guidelines advocated active patient identification even in the absence of a consensus about the best approach [10]. The World Health Organization has defined osteoporosis using a T-score cut-off, and BMD assessments were initially the primary tool for patient identification. More recently, other independent clinical risk factors (in addition to BMD) have been shown to enhance the efficiency of identifying candidates for therapy [11], and patient identification approaches have been broadened to include these other factors. 

The FRAX tool, for example, was designed to help physicians in clinical practice to identify patients who are at high risk of fracture by estimating the 10-year fracture risk for an individual compared with a population of the same age and sex [12]. The recent incorporation of FRAX into the US osteoporosis screening guidelines acknowledges the importance of assessing risk factors beyond BMD and defines a routine role for FRAX in the primary care setting to identify women (and men) who would be most likely to benefit from osteoporosis therapy [13, 14]. FRAX is also included in osteoporosis guidelines for a number of countries outside of the USA [15, 16]. For example, Canada has adopted FRAX to identify candidates for osteoporosis therapy and also has created a customized version using the Canadian national hip fracture and mortality data [17, 18]. The use of FRAX in clinical practice has also been shown to improve prescribing practices for osteoporosis therapies [19]. Although, we found no association between on-study fracture experience and NOF FRAX treatment thresholds among subjects on nonpharmacologic therapy, we did observe a greater incidence of on-study fractures among pharmacologically treated subjects who met or exceeded the treatment thresholds compared with subjects whose scores were below the treatment thresholds.

This shift beyond using only BMD assessment to identify individuals at increased risk of fracture may, in part, also reflect concerns about inadequate access to dual energy X-ray absorptiometry (DXA) in Europe and the USA [11, 20], as well as increased understanding of the role of other risk factors. Recent estimates suggest that most European countries lack the DXA resources required for case finding and treatment monitoring, and in the USA, there is concern that reductions in Medicare reimbursements for DXA will result in the underuse of BMD assessments in Medicare populations [11, 20]. 

By 2025, an estimated 3 million osteoporotic fractures are projected to occur in the USA, and these fractures are associated with an increased risk of subsequent fractures, significant treatment costs, quality of life decrements, and increased mortality risk [21–25]. In the USA and elsewhere, a variety of effective therapies with different modes of administration and mechanisms of action are available for both the prevention and treatment of osteoporosis [26]. The combination of the large public health burden associated with osteoporosis-related fracture and the availability of effective osteoporosis therapies suggests that there may be substantial benefit from identifying individuals at greatest risk for fracture and providing those individuals with appropriate therapeutic interventions [26]. The results of our study support the current published recommendations to evaluate a variety of fracture risk factors, and suggest that, in addition to BMD, age, history of fracture, and smoking status may be the most common risk factors in postmenopausal women identified by primary care physicians for osteoporosis therapy.

Various limitations must be considered in interpreting the results of this study. On-study fracture was identified using subject-reported data. Therefore, the accuracy of these data may be affected by the use of a 6-month recall window and by other factors limiting subject recall. The physician-reported diagnoses used to characterize and group subjects may have been based on clinical judgment, in addition to explicit risk factors. This may explain why some women who were reported to have no risk factors in our analysis were actually receiving treatment. Although the women in this cohort are demographically similar to women treated for osteoporosis in the USA [5], they may not be representative of women with postmenopausal bone loss overall or those treated for bone loss in countries outside of the USA. In addition, in the time since the POSSIBLE US data were collected, evidence highlighting risks and limitations of bisphosphonate therapies has been published [27, 28]. In light of these findings, primary care physicians may have become less likely to recommend osteoporosis treatment for low risk patients. The utility of FRAX scores computed for the women in POSSIBLE US cohort is still being assessed because FRAX was initially developed to predict fracture risk only in untreated individuals. In this context, the “untreated” population includes individuals who have no pharmacological treatment history, as well as individuals who used oral bisphosphonates for <2 months in the previous 2 years and individuals with no estrogen, raloxifene, calcitonin, or denosumab use in the past year but who may have had prior use of these agents [29]. However, a recent validation study using 5 years of follow up data from a large Canadian population-based cohort concluded that FRAX predicted the risk of major osteoporotic and hip fractures equally well in untreated, currently treated, and previously treated women [8]. The authors note that this finding should not be interpreted as meaning that osteoporosis treatment is ineffective, and although their study was not powered to detect an antifracture benefit of therapy, the actual fracture experience in the most adherent patient group was lower than the one predicted by FRAX. Finally, there are a few data limitations which may impact the FRAX scores. BMD assessments were not required by the POSSIBLE US study protocol; 11.1% of subjects in the osteoporosis group and 7.6% of subjects in the osteopenia group did not have T-scores for use in the FRAX calculation. Also, by defaulting the alcohol use and secondary osteoporosis diagnosis risk factors to “no” in the FRAX calculator, we may have underestimated the 10-year predicted fracture risks for some subjects.

In summary, our study highlighted that multiple fracture risk factors were present in a significant proportion of the postmenopausal women with osteoporosis receiving treatment. Multiple risk factors were associated with a greater incidence of on-study fracture (overall and osteoporosis-related fractures), even though the women in this study had been identified by their primary care physicians to receive osteoporosis therapy during the followup period. By demonstrating the association between the presence of multiple risk factors and fracture incidence, this study underscores the importance of considering independent fracture risk factors (beyond BMD) to identify postmenopausal women who could benefit from osteoporosis treatments. These results also suggest that women with multiple risk factors may remain at elevated risk of fracture even after initiating therapy.

## Figures and Tables

**Table 1:** Subject-reported on-study fracture.

Subjects with	Osteoporosis group (*N* = 1,916)	Osteopenia group (*N* = 2,513)
	*n* (%)	*n* (%)
No on-study fracture	1,692 (88.3)	2,284 (90.9)
Any on-study fracture	224 (11.7)	229 (9.1)
Any osteoporosis- related fracture	153 (8.0)	138 (5.5)
Hip fracture	35 (1.8)	22 (0.9)
Spine fracture	35 (1.8)	29 (1.2)
Nonhip/nonspine fracture	104 (5.4)	99 (3.9)
Multiple osteoporosis- related fractures	46 (2.4)	37 (1.5)

**Table 2:** Subject-reported on-study osteoporosis-related fracture stratified by number of risk factors.

	Subjects with 0 risk factors	Subjects with 1 risk factor	Subjects with ≥2 risk factors
	*n* (%)	*n* (%)	*n* (%)
Osteoporotic subjects	214	663	1,039
Any osteoporosis-related fracture	9 (4.2)	41 (6.2)	103 (9.9)
Hip fracture	0 (0)	4 (0.6)	31 (3.0)
Spine fracture	2 (0.9)	7 (1.1)	26 (2.5)
Nonhip/nonspine fracture	7 (3.3)	33 (5.0)	64 (6.2)
Multiple osteoporosis-related fractures	1 (0.5)	10 (1.5)	35 (3.4)

Osteopenic subjects	1,322	843	348
Any osteoporosis-related fracture	59 (4.5)	40 (4.8)	39 (11.2)
Hip fracture	8 (0.6)	6 (0.7)	8 (2.3)
Spine fracture	11 (0.8)	7 (0.8)	11 (3.2)
Nonhip/nonspine fracture	47 (3.6)	28 (3.3)	24 (6.9)
Multiple osteoporosis-related fractures	14 (1.1)	10 (1.2)	13 (3.7)

Risk factors: age > 70 years, history of fracture since age 50, minimum hip or spine T-score ≤ −2.5 at diagnosis, body mass index < 18.5 kg/m^2^, rheumatoid arthritis, parental history of hip fracture, current cigarette smoking, and glucocorticoid use in 6 months prior to study entry.

**Table 3:** Self-reported on-study fracture among nonpharmacologically treated^a^ POSSIBLE US subjects stratified by the National Osteoporosis Foundation treatment thresholds.

	Number of subjects with FRAX score	FRAX predicted 10-year risk of ≥3% for hip fracture			FRAX predicted 10-year risk of ≥20% for major osteoporotic fracture			FRAX score ≥ intervention threshold for either hip or major osteoporotic fractures		
	(*N* = 1,299) *n* (%)	Yes (*N* = 407) *n* (%)	No (*N* = 892) *n* (%)	*P* value	Yes (*N* = 206) *n* (%)	No (*N* = 1,093) *n* (%)	*P* value	Yes (*N* = 411) *n* (%)	No (*N* = 888) *n* (%)	*P* value
No on-study fracture	1,227 (94.5)	384 (94.4)	843 (94.5)	0.91	192 (93.2)	1,035 (94.7)	0.39	388 (94.4)	839 (94.5)	0.95
Any on-study fracture	72 (5.5)	23 (5.7)	49 (5.5)		14 (6.8)	58 (5.3)		23 (5.6)	49 (5.5)	
Any osteoporosis- related fracture	47 (3.6)	18 (4.4)	29 (3.3)	0.29	12 (5.8)	35 (3.2)	0.06	18 (4.4)	29 (3.3)	0.33
Hip fracture	9 (0.7)	3 (0.7)	6 (0.7)	0.90	1 (0.5)	8 (0.7)	0.70	3 (0.7)	6 (0.7)	0.91
Spine fracture	9 (0.7)	4 (1.0)	5 (0.6)	0.40	4 (1.9)	5 (0.5)	0.02	4 (1.0)	5 (0.6)	0.41
Nonhip/non- spine fracture	31 (2.4)	11 (2.7)	20 (2.2)	0.61	7 (3.4)	24 (2.2)	0.30	11 (2.7)	20 (2.3)	0.64
Multiple osteoporosis- related fractures	11 (0.9)	6 (1.5)	5 (0.6)	0.10	4 (1.9)	7 (0.6)	0.06	6 (1.5)	5 (0.6)	0.10

^a^Subjects who reported no pharmacological therapy or using only calcium/vitamin D within 2 months of study entry.

*P* value is for pairwise (yes/no) comparisons with percentage of specified fracture risk outcome for each risk category.

**Table 4:** Self-reported on-study fracture among pharmacologically treated^a^ POSSIBLE US subjects stratified by the National Osteoporosis Foundation treatment thresholds.

	Number of subjects with FRAX score	FRAX predicted 10-year risk of ≥3% for hip fracture			FRAX predicted 10-year risk of ≥20% for major osteoporotic fracture			FRAX score ≥ intervention threshold for either hip or major osteoporotic fractures		
	*N* = 2,996 *n* (%)	Yes (*N* = 1,039) *n* (%)	No (*N* = 1,957) *n *(%)	*P*-value	Yes (*N* = 610) *n* (%)	No (*N* = 2,386) *n* (%)	*P*-value	Yes (*N* = 1,062) *n* (%)	No (*N* = 1,934) *n* (%)	*P*-value
No on-study fracture	2,623 (87.6)	865 (83.3)	1,758 (89.8)	<0.0001	492 (80.7)	2,131 (89.3)	<0.0001	885 (83.3)	1,738 (89.9)	<0.0001
Any on-study fracture	373 (12.5)	174 (16.8)	199 (10.2)		118 (19.3)	255 (10.7)		177 (16.7)	196 (10.1)	
Any osteoporosis- related fracture	240 (8.0)	126 (12.1)	114 (5.8)	<0.0001	93 (15.3)	147 (6.2)	<0.0001	127 (12.0)	113 (5.8)	<0.0001
Hip fracture	48 (1.6)	34 (3.3)	14 (0.7)	<0.0001	30 (4.9)	18 (0.8)	<0.0001	35 (3.3)	13 (0.7)	<0.0001
Spine fracture	54 (1.8)	32 (3.1)	22 (1.1)	0.0001	29 (4.8)	25 (1.1)	<0.0001	32 (3.0)	22 (1.1)	0.0002
Nonhip/non- spine fracture	169 (5.6)	79 (7.6)	90 (4.6)	0.0007	52 (8.5)	117 (4.9)	0.0005	80 (7.5)	89 (4.6)	0.0009
Multiple osteoporosis- related fractures	72 (2.4)	41 (4.0)	31 (1.6)	0.0001	34 (5.6)	38 (1.6)	<0.0001	42 (4.0)	30 (1.6)	<0.0001

^a^Subjects who reported the use of pharmacological monotherapy or combination therapy at study entry or initiated pharmacological therapy within 2 months of entry.

*P* value is for pairwise (yes/no) comparisons with percentage of specified fracture risk outcome for each risk category.

## References

[1] WHO Collaborating Centre for Metabolic Bone Diseases FRAX: WHO risk assessment tool. http://www.sheffield.ac.uk/FRAX.

[2] McCloskey E FRAX: identifying people at high risk of fracture. http://www.osteoporosis.org.za/downloads/FRAX-report-09.pdf.

[3] U.S. Preventive Services Task Force (2011). Screening for osteoporosis: U.S. Preventive Services Task Force recommendation statement. *Annals of Internal Medicine*.

[4] National Osteoporosis Foundation (2010). *Clinician's Guide To Prevention and Treatment of Osteoporosis*.

[5] Barrett-Connor E, Ensrud K, Tosteson ANA (2009). Design of the POSSIBLE US Study: postmenopausal women’s compliance and persistence with osteoporosis medications. *Osteoporosis International*.

[6] Warriner AH, Patkar NM, Curtis JR (2011). Which fractures are most attributable to osteoporosis?. *Journal of Clinical Epidemiology*.

[7] Tosteson ANA, Do TP, Wade SW, Anthony MS, Downs RW (2010). Persistence and switching patterns among women with varied osteoporosis medication histories: 12-month results from POSSIBLE US. *Osteoporosis International*.

[8] Leslie WD, Lix LM, Johansson H, Oden A, McCloskey E, Kanis JA (2012). Does osteoporosis therapy invalidate FRAX for fracture prediction?. *Journal of Bone and Mineral Research*.

[9] Geusens P (2009). Strategies for treatment to prevent fragility fractures in postmenopausal women. *Best Practice and Research*.

[10] Geusens PP (2003). Review of guidelines for testing and treatment of osteoporosis. *Current Osteoporosis Reports*.

[11] De Laet C, Odén A, Johansson H, Johnell O, Jönsson B, Kanis JA (2005). The impact of the use of multiple risk indicators for fracture on case-finding strategies: a mathematical approach. *Osteoporosis International*.

[12] Siris ES, Baim S, Nattiv A (2010). Primary care use of FRAX: absolute fracture risk assessment in postmenopausal women and older men. *Postgraduate Medicine*.

[13] Kanis JA, McCloskey EV, Johansson H, Oden A (2009). Approaches to the targeting of treatment for osteoporosis. *Nature Reviews*.

[14] Kanis JA, McCloskey EV, Johansson H, Oden A, Ström O, Borgström F (2010). Development and use of FRAX in osteoporosis. *Osteoporosis international*.

[15] Kanis JA, Burlet N, Cooper C (2008). European guidance for the diagnosis and management of osteoporosis in postmenopausal women. *Osteoporosis International*.

[16] http://www.shef.ac.uk/NOGG/.

[17] Leslie WD, Lix LM, Langsetmo L (2011). Construction of a FRAX model for the assessment of fracture probability in Canada and implications for treatment. *Osteoporosis International*.

[18] Papaioannou A, Morin S, Cheung AM (2010). 2010 clinical practice guidelines for the diagnosis and management of osteoporosis in Canada: Summary. *Canadian Medical Association Journal*.

[19] Leslie WD, Morin S, Lix LM (2010). A before-and-after study of fracture risk reporting and osteoporosis treatment initiation. *Annals of Internal Medicine*.

[20] King AB, Fiorentino DM (2011). Medicare payment cuts for osteoporosis testing reduced use despite tests' benefit in reducing fractures. *Health Affairs*.

[21] Burge R, Dawson-Hughes B, Solomon DH, Wong JB, King A, Tosteson A (2007). Incidence and economic burden of osteoporosis-related fractures in the United States, 2005–2025. *Journal of Bone and Mineral Research*.

[22] Center JR, Bliuc D, Nguyen TV, Eisman JA (2007). Risk of subsequent fracture after low-trauma fracture in men and women. *Journal of the American Medical Association*.

[23] Dempster DW (2011). Osteoporosis and the burden of osteoporosis-related fractures. *The American Journal of Managed Care*.

[24] Ioannidis G, Papaioannou A, Hopman WM (2009). Relation between fractures and mortality: results from the Canadian Multicentre Osteoporosis Study. *Canadian Medical Association Journal*.

[25] Strom O, Borgstrom F, Kanis JA (2011). Osteoporosis: burden, health care provision and opportunities in the EU: a report prepared in collaboration with the International Osteoporosis Foundation (IOF) and the European Federation of Pharmaceutical Industry Associations (EFPIA). *Archives of Osteoporosis*.

[26] Rachner TD, Khosla S, Hofbauer LC (2011). Osteoporosis: now and the future. *The Lancet*.

[27] Black DM, Bauer DC, Schwartz AV, Cummings SR, Rosen CJ (2012). Continuing bisphosphonate treatment for osteoporosis: for whom and for how long?. *The New England Journal of Medicine*.

[28] Whitaker M, Guo J, Kehoe T, Benson G (2012). Bisphosphonates for osteoporosis: where do we go from here?. *The New England Journal of Medicine*.

[29] http://www.iscd.org/resources/fracture-risk-models/.

