# Corrigendum to “Prevalence of Fracture Risk Factors in Postmenopausal Women Enrolled in the POSSIBLE US Treatment Cohort”

**DOI:** 10.1155/2017/4827469

**Published:** 2017-10-25

**Authors:** Nicole Yurgin, Sally Wade, Sacha Satram-Hoang, David Macarios, Marc Hochberg

**Affiliations:** ^1^Amgen Inc., One Amgen Center Drive, Thousand Oaks, CA 91320-1799, USA; ^2^Wade Outcomes Research and Consulting, 358 South 700 East, Suite B432, Salt Lake City, UT 84102, USA; ^3^Q.D. Research, Inc., 8789 Auburn Folsom Road, Suite C501, Granite Bay, CA 95746, USA; ^4^Division of Rheumatology and Clinical Immunology, University of Maryland School of Medicine, 10 S. Pine Street, MSTF 8-34, Baltimore, MD 21201, USA

In the article titled “Prevalence of Fracture Risk Factors in Postmenopausal Women Enrolled in the POSSIBLE US Treatment Cohort” [1], there was an error regarding the FRAX® tool, which should be clarified as follows:

The article notes “The WHO fracture risk assessment tool (FRAX) is a computer-based algorithm that assesses fracture probability of men and women [1].” However, the World Health Organization (WHO) did not develop, test, or endorse the FRAX tool or its recommendations [2]. The metabolic bone disease unit at the University of Sheffield that developed FRAX was a WHO Collaborating Centre from 1991 to 2010, but treatment guidelines must undergo a formal process before they can be endorsed by the WHO.
